# *PiggyBac* Mediated Multiplex Gene Transfer in Mouse Embryonic Stem Cell

**DOI:** 10.1371/journal.pone.0115072

**Published:** 2014-12-17

**Authors:** Xibin Lu, Wei Huang

**Affiliations:** 1 Department of Biochemistry, the University of Hong Kong, Hong Kong, China; 2 Department of Biology, Shenzhen Key Laboratory of Cell Microenvironment, South University of Science and Technology of China, Shenzhen, China; National University of Singapore, Singapore

## Abstract

*PiggyBac* system has been shown to have a high efficiency to mediate gene transfer. However, there are no reports on its efficiency to mediate multiplex transgenes in mouse embryonic stem cells. Here we first established an immortalized feeder cell line by introducing four antibiotic resistance genes simultaneously into the original SNL 76/7 feeder cell line utilizing the *PiggyBac* system. This is the feeder cell line with the most diverse types of antibiotic resistance genes reported so far, which will enable researchers to perform simultaneous multiplex gene transfer or gene targeting experiments in ES cells. With such feeder cell line, we were able to quantitatively characterize the transposition efficiency of *PiggyBac* system in mouse ES cells using five transposons carrying different inducible fluorescence proteins and antibiotic resistance genes, and the efficiency ranged from about 2% for one transposon to 0.5% for five transposons. The highly efficient multiplex gene transfer mediated by *PiggyBac* will no doubt provide researchers with more choices in biomedical research and development.

## Introduction

Embryonic stem cells (ESCs) are derived from the inner cell mass (ICM) of preimplantation blastocyst in many species [1], [2]. They can go through numerous cell divisions while maintain undifferentiated state, a phenomenon called self-renewal. In addition, ESCs have the ability to differentiate into a wide variety of cell types both *in vitro* and *in vivo*. *In vitro* ESCs are usually cultured on mouse embryonic fibroblasts (MEFs) feeder layers which are derived from day12.5–14.5 mouse embryos. MEFs can secret growth factors to support ES cell growth and Leukemia Inhibitory Factor (LIF) to prevent ES cell differentiation. However, MEFs have relatively short lifetime and have to be isolated from mice repeatedly. This process is often time-consuming and costly. Compared to the commonly used primary MEFs, SNL 76/7 feeder cells [3], which were derived from a STO cell line, are also widely used as feeder layers. The SNL 76/7 feeder cells are stably transfected with a neomycin resistance gene and LIF gene. It has one striking advantage for indefinite propagation. And it has been widely used for mouse and human ES cell culture as well as induced pluripotent stem cell (iPSC) maintenance [4]–[6].

Currently, MEFs are mainly used for routine maintenance of ES cell culture. It also plays important role in gene targeting experiments involving the selection of antibiotic resistance stable clones in transfected ES cells. Antibiotic resistance MEFs are usually derived from transgenic mice and neomycin, hygromycin or puromycin resistance MEFs have been successfully established [7]–[9]. Tucker *et al.* established a DR4 transgenic strain which was resistant to hygromycin, G418, puromycin as well as 6TG simultaneously [10], and this is the established mouse strain with most antibiotic resistance markers reported so far. Luchi *et al.* established an immortalized blasticidin and zeocin resistance cell line which was used for the propagation of human ESCs [11]. However, researchers occasionally need to transfect several cassettes with multiple antibiotic resistance markers into ESCs simultaneously. Derivation of such MEFs from transgenic mouse strain involves repeated mice breeding and time-consuming cell isolation. Furthermore, the established DR4 MEFs may not satisfy research needs in many demanding situations. Therefore, it is imperative to establish such a feeder cell line using an alternative method.

The *PiggyBac* (PB) transposon was first discovered by Fraser *et al.* from the cabbage looper moth *Trichoplusia ni* [12], and then it was isolated by Cary *et al.* in 1989 [13]. Later, it was found to have high transposition efficiency across different species. Ding *et al.* demonstrated that PB is very efficient for genetic manipulation including transgenesis and insertional mutagenesis in mice and other vertebrates [14]. Compared with *Sleeping Beauty* or *Tol*2 transposons, PB has higher transposition activity in mammalian cells [15]. Another striking advantage for PB is that it has high cargo capacity (up to 100 kb) compared with virus mediated gene transfer [16]. In addition, integrated transgenes could be excised without “footprint” [17]. Although there are numerous reports on its high efficiency mediating single gene transfer, there are few reports on its efficiency for multiplex genes transfer, especially in mouse ESCs.

In this study, we first established an immortalized SNL feeder cell line (SNL DG5) in which four additional antibiotic resistance genes were transfected into SNL 76/7 feeder cells simultaneously utilizing the aforementioned *PiggyBac* system. Totally five antibiotics resistance genes that confer hygromycin^R^, puromycin^R^, blasticidin^R^, zeocin^R^ and G418^R^ coexisted. In addition, we quantitatively measured *PiggyBac* mediated transposition efficiency on multiplex gene transfer in mouse ESCs using multiplex inducible fluorescence reporters for the first time.

## Materials and Methods

### Materials

For molecular cloning, all restriction enzymes, T4 DNA polymerase and T4 DNA ligase are from NEB (Ipswich, MA, USA). For mammalian cell culture, DMEM, common FBS, ES cell qualified FBS are from Invitrogen (Carlsbad, CA, USA). Antibiotics used for stable cell selection are from Invitrogen and Sigma (St Louis, MO, USA). CCE cells [18], [19], a mouse ES cell line, were a gift from Stem Cell Technologies (Vancouver, BC, Canada). The mAmetrine and tdTomato FPs are subcloned from Addgene plasmid 18879 [20]. All other FPs are from Clontech (Mountain View, CA, USA).

### Vector construction

PL451 plasmid was used as the original backbone. HS4 insulator was amplified from plasmid pEGFP-N1-Cha4 (gift from prof. Chiju Wei) which contains two tandem repeats of core cHS4. The 235 bp 5′ terminal repeat and 313 bp 3′ terminal repeat of *PiggyBac* transposon were amplified from the plasmid PB-SB-Neo (gift from Prof. Pentao Liu). HS4 insulator was first inserted into *Eco*R I and *Nhe* I site of PL451. Then the 5′ terminal repeat and HS4 insulator were cloned into the *Kpn* I and *Apa* I sites using three-piece ligation. The 3′ terminal repeat and HS4 insulator were cloned into *Not* I and *Bst*x I sites using the same method. The constructed plasmid was named as **PB5-HS4-MCS-HS4-PGk-Neo-GpA-HS4-PB3 (pBX-023)**. The puromycin, hygromycin, blasticidin and zeocin resistance genes were amplified from pIRES-puro3, pSilencer3.1-H1, pcDNA6/TR and pcDNA4/TO, respectively, and then were cloned into pEGFPnuc-GpA plasmid in which the EGFPnuc cassette was replaced by antibiotics resistance gene cassettes. Finally, the EF1α promoter was amplified from pEF plasmid and cloned in front of each antibiotics resistance genes to make pEF1α-drug^R^-GpA (drug^R^ stands for different antibiotics resistance encoding genes resistant to neomycin, hygromycin, puromycin, zeocin or blasicidin). The assembled cassette was finally cloned into pBX-023 to create different antibiotic resistance vectors **PB5-HS4-EF1α-drug^R^-GpA-HS4-PB3.** To generate tetracycline response element (TRE) driven inducible fluorescence protein constructs, we first established a series of plasmids **PL-MCS-FPs-nuc-2A-drug^r^-GpA** (FPs represent different fluorescent proteins including EGFP, mAmetrine, CFP, mCherry and tdTomato; nuc represents three repeats of nuclear localization signals, 2A represents *Thoseaasigna* virus 2A “self-cleaving” peptide [21]), then TRE promoter was amplified from pTRE-tight template and cloned into the multiple cloning sites which constitute PL-TRE-FPs-nuc-2A-drug^R^-GpA plasmids. Followed by digestion with *Age* I and *Not* I and cloned into pBX-023 to get a series of **PB5-HS4-TRE-FPs-nuc-T2A-drug^R^-GpA-HS4-PB3** cassettes. The transactivator rtTA expressing cassette was constructed using sequence and ligation-independent cloning (SLIC) method [22]. Specifically, separate SV40 promoter, puro, 2A-rtTA and SV40 pA carrying overlap sequences were PCR amplified using corresponding template and then were treated with T4 DNA polymerase. *Xho* I and *Eco*R I digested pBX-023 was also treated with T4 DNA polymerase. After treatment, equimolar amounts of vector and inserts were mixed and incubated at 37°C for 30 minutes. The correct transformant was named as **PB5-HS4-SV40-puro-2A-rtTA-pA-HS4-PGk-Neo-GpA-HS4-PB3 (pBX-092)**.

### Cell culture and transfection

The SNL 76/7 feeder cell [3] (gift from Dr. Martin Cheung) was cultured with DMEM supplemented with 10% FBS and 1% (v/v) penicillin-streptomycin (Invitrogen). When cell reaches about 60%–70% confluence, it was co-transfected with 1 µg (per 35 mm dish) plasmids of each of four independent transposon carrying antibiotic resistance genes and PGK-transposase using lipofectamine 2000 according to the manufacturer’s protocol. The ratio of transposon to transposase plasmids is 2∶1. Cells stably expressing four antibiotic resistance genes were selected for 10 days in growth medium supplemented with 80 µg/mL zeocin, 200 µg/mL hygromycin, 4 µg/mL puromycin and 6 µg/mL blasticidin concentration which was high enough to support ES cell gene targeting applications. We also used higher concentrations of antibiotics, including 400 µg/mL zeocin, 1000 µg/mL hygromycin, 20 µg/mL purocin and 30 µg/mL blasticidin to select high resistance cell population. After 10 days, resistant cells were trypsinized and transferred in 10 cm dish for propagation.

For stable transfection of TRE-FPs-2A-drug^R^ and SV40-rtTA transposons, the aforementioned multiple antibiotics resistance SNL feeder cells (mitomycin-C treated) were first seeded in 6-well plate with appropriate density. The next day, CCE ES cell line was cultured with high glucose DMEM with 15% ES-qualified FBS as well as 1000 U/mL LIF, 50 units/mL Penicillin and 50 µg/mL Streptomycin, 0.1 mM 1-thiglycerol, 0.1 mM non-essential amino acid, 2 mM Gluta^MAX^ (Invitrogen) according to the standard protocol. When ES cells reach about 40%–50% confluence, 0.7 µg plasmids of each transposon and the transposase were mixed and transfected into CCE ES cells using lipofectamine 2000. The transfection method was similar with aforementioned. After 7–8 days of selection using different antibiotics combinations, ES cell clones were observed under fluorescence microscopy, handpicked from five antibiotics selection well and propagated for future genotyping and flow cytometry analysis.

### Genotyping analysis

Transfected SNL DG5 feeder cells and CCE ES cells were cultured in 24-well plate. The plate for CCE ES cell culture was first coated with 0.1% gelatin. When cells reach about 60–70% confluence, cells were trypsinized and washed twice with PBS, treated with lysis buffer and proteinase K overnight at 56°C water bath. The next day, genomic DNA was isolated and dissolved in ddH2O. Subsequently, 1 µl of DNA was used as template for PCR amplification. PCR was performed with 30 cycles of 94°C 30 s, 58°C 30 s, and 72°C 60 s.

### Antibiotic resistance assay

Antibiotic resistance test was assayed by seeding 2×10^4^ mytomycin-treated cells in each well of 96-well plate and adding puromycin, hygromycin, zeocin and blasticidin with different concentrations. The medium containing corresponding antibiotics was changed every other day. After 7 days treatment, the cell viability was assayed using MTT assay by adding 10 µl/well of 5 mg/mL MTT (sigma) into 100 µl medium, after 3–4 hours, 20 µl lysis buffer (20% SDS+50% DMF) was added into each well and incubated at 37°C for overnight. The next day, measurement of the spectrophotometrical absorbance was performed at 570 nm wavelength.

### Alkaline Phosphotase (AP) staining for calculating transposition efficiency

For calculating transposition efficiency of inducible TRE-FPs-2A-drug^R^ stable clones, the medium in each well was removed and washed twice with PBS. Then cells were fixed with 4% PFA for 10 minutes and washed 2 or 3 times with 1xPBS. The fixed cells were stained with AP substrate solution (System Biosciences, USA) for 10 minutes and followed by washing with 1xPBS for 3–4 times. The exact ES cell clone numbers were counted manually with the enlarged printouts.

### Image processing and flow cytometry analysis

To verify the quality of our newly established SNL DG5 feeder cell, we compared SNL DG5 feeder cell with commonly used MEF feeder cell (derived from embryo day 13.5). Specifically, we used two ES reporter cell line: NG2S (Nanog-EGFP reporter cell lines from our lab) and RGOC (Rex-1-GFP reporter [23]). SNL DG5 and MEF were seeded into 6-well plate (0.1% gelatin coated) with the density 5×10^4^ cells per cm^2^. The next day, NG2S and RGOC were seeded in the feeder plated wells with appropriate cell numbers. The two ES cell lines were cultured on the aforementioned feeders for three consequent passages before analysis. Bright-field images were taken with Nikon TE2000 microscopy and flow cytometry analysis with BD FACSCantoII Analyzer (488 blue laser). For flow analysis of ES cell lines carrying multiple fluorescent proteins under the control of tetON expression system, each clone was split into 2 wells of 12-well plate, 1 µg/mL of doxycline was added into one well and another well was used as negative control. Three days after doxycline induction, cells were trypsinized and neutralized by DMEM supplemented with 10% FBS, and analyzed using BD LSR Fortessa Analyzer with three lasers (488 nm, 405 nm and 561 nm).

### Statistical analysis

Calculations of the mean and CV% were performed using Microsoft Excel. *T-*test was performed on the raw data obtained. A value of P<0.05 was considered statistically significant.

## Results

### Generation of transposable constructs with different antibiotic resistance genes

**Figure 1:**
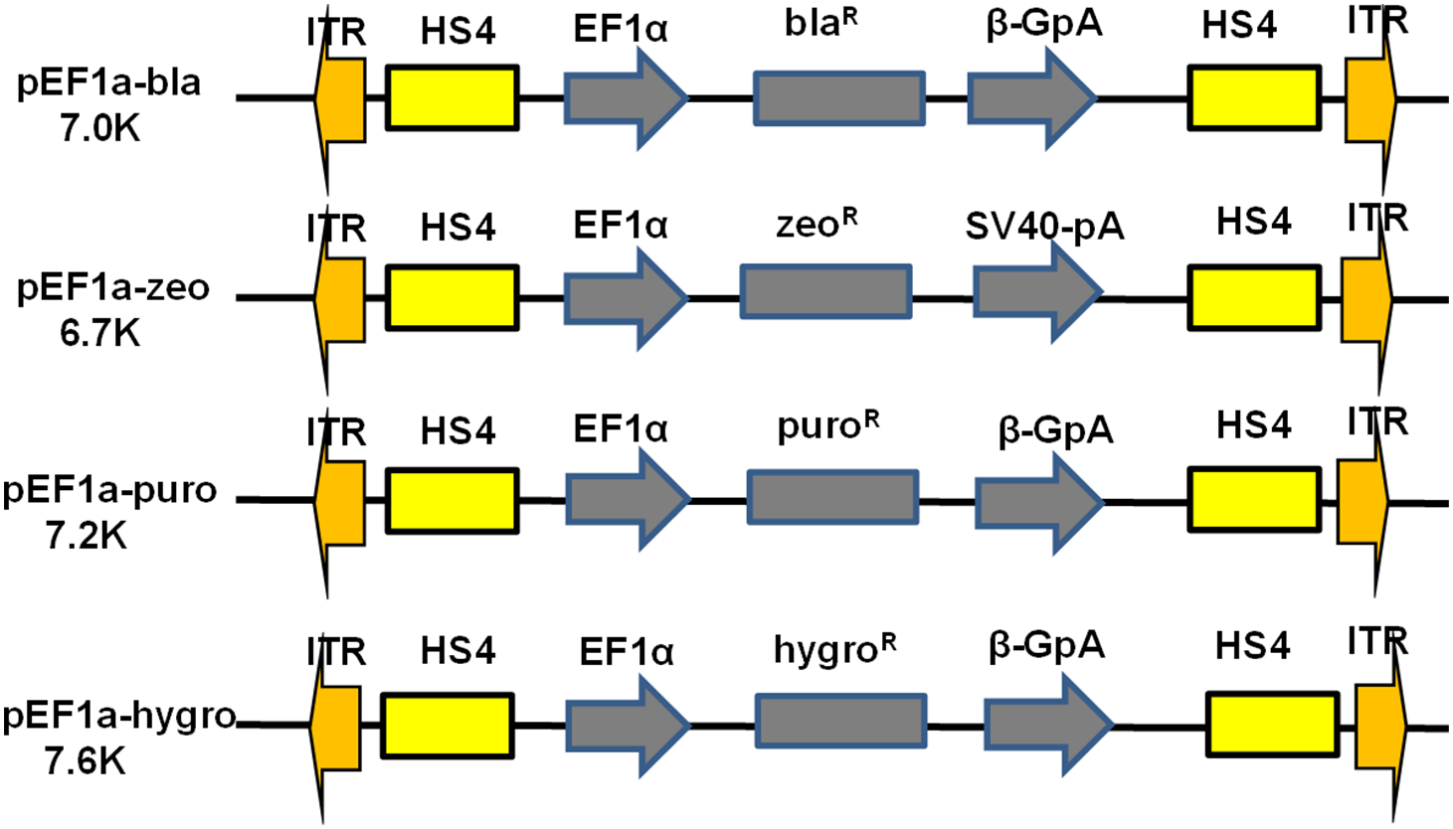
Schematic representation of four antibiotic resistance gene expressing transposons based on *PiggyBac* backbone. Bla^R^, blasticidin-resistance gene; zeo^R^, zeocin-resistance gene; puro^R^, puromycin resistance gene; hygro^R^, hygromycin resistance gene; EF1α, human elongation factor 1α promoter; ITR, terminal repeats; HS4, chicken β-globin hypersensitive site 4 insulator.

Based on previous studies, a 5′ terminal repeat of 235 bp and a 3′ terminal repeat of 313 bp *PiggyBac* transposon were used in this study. Two copies of core chicken β-globin hypersensitive site 4 insulator (cHS4) was also flanked the DNA transposon to minimize surrounding chromosomal effect. Previous studies showed that EF1α promoter was relatively strong and less susceptible to post-integrative gene silencing [24], [25]. Therefore, it was introduced in our constructs to drive constitutive expression of antibiotic resistance genes (**Fig. 1**).

### Establishment of SNL DG5 feeder cell line

**Figure 2:**
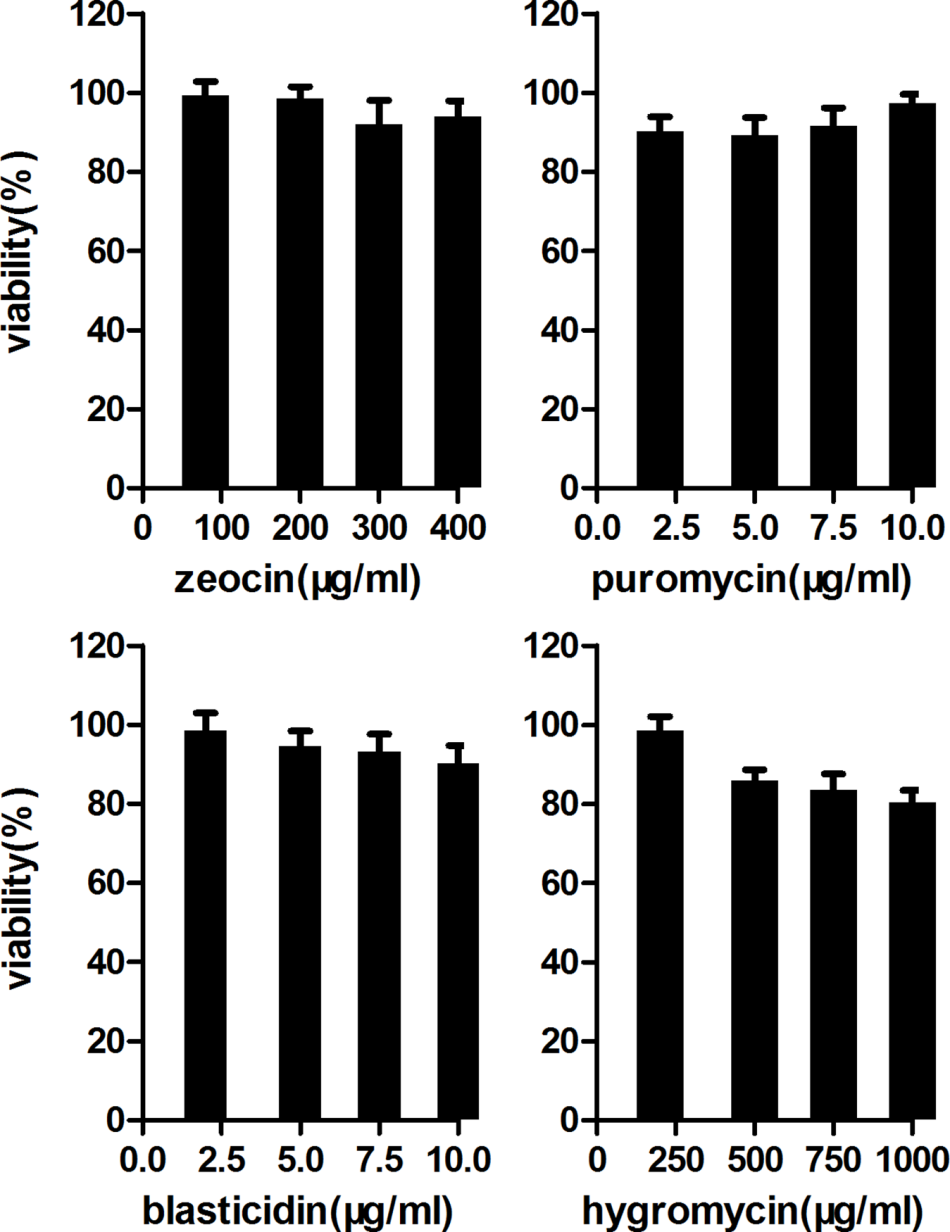
Antibiotic resistance of mitomycin treated SNL DG5 feeder layer. About 2×10^4^ mitomycin C-treated SNL DG5 feeder cells were plated in each well of 96-well plate. The medium was added with different antibiotics concentrations and each concentration was in quadruplicate. Medium was changed every two days and the cells were cultured for 7 days. Survival rate is calculated by comparing to parallel-plated cells without antibiotics selection and expressed as a percentage.

In order to introduce multiple plasmids into SNL feeder cell, we adopted the *PiggyBac* transposon system that has been demonstrated to be highly efficient to transfer foreign DNA in mouse ESCs [26]. We transfected four independent transposons together with a PGK-driven transposase expression plasmid into the SNL feeder cell simultaneously. Genotyping analysis showed that four transgene cassettes have been successfully integrated into the genome (**S1 Figure**). To establish feeder cell populations with low and high antibiotics concentrations, two and ten folds of the typical ES cell antibiotics selection concentrations (40 µg/mL zeocin, 100 µg/mL hygromycin, 2 µg/mL puromycin and 3 µg/mL blasticidin) were used separately. MTT experiment (**Fig. 2**) shows that after seven days of incubation with different antibiotics concentrations, the mitotically-inactivated (mitomycin C) SNL DG5 feeder cells maintains close to 100% viability which is sufficient to support general ES cell selection.

### Growth curve of established feeder cell line

**Figure 3:**
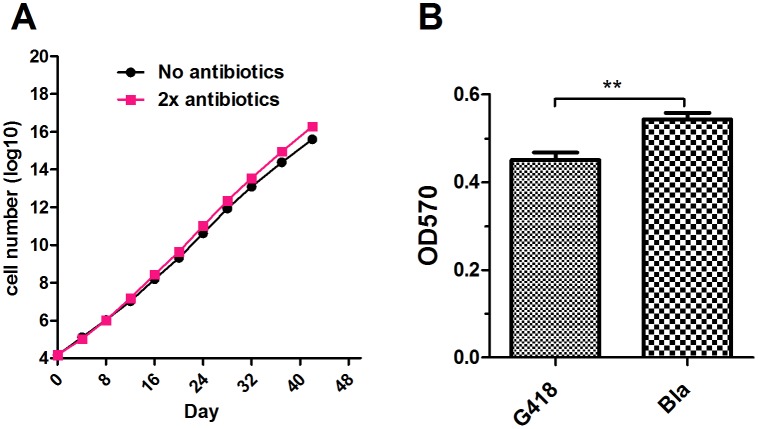
Stable transgene expression of established SNL DG5 feeder cell. (A) The cells were seeded into 24-well plate and each was in duplicate. Antibiotics or antibiotics-free medium were added into each well for cell culture. The 2x antibiotics concentration was 80 µg/mL zeocin, 200 µg/mL hygromycin, 4 µg/mL puromycin and 6 µg/mL blasticidin respectively. The cell numbers were calculated every four days during each subculture and identical numbers of cells were seeded for continuous passaging. (B) MTT assay for comparison of *PiggyBac* mediated blasticidin resistance gene with original G418 resistance gene expression after long time cell culture.

Transgene silencing is an unavoidable phenomenon in random stable transfection experiments. Although *PiggyBac* was shown to mediate long time transgene expression both *in vitro* and *in vivo*, it still showed transgene silencing in different cell lines [25], [27]. To validate the transgene expression stability of the established feeder cell over long period of time, the SNL DG5 feeder cell line was passaged continuously with or without antibiotics selection pressure and the population amplifications were measured at every passage. If transgene silencing happened in cell, it would not survive the antibiotics selection pressure. The consequence at population level would be that the apparent cell population growth would be slower under antibiotics selection pressure. For the antibiotics selection pressure group, we adopted two-fold concentrations to maintain cell culture continuously. The medium was changed every four days. After the extended culture for 42 days, the SNL DG5 cells are still capable of propagating exponentially with compatible growth rates (**Fig. 3A**). This result indicates that the introduced transgenes can maintain expression without silencing. Since HS4 insulator was flanked in both sides of transgenes, to test HS4 insulation effect on transgene expression stability, we compared the transgene expressions between blasticidin and G418 resistance gene which already existed in original SNL 76/7 feeder cell line. After 42-day continuous cell culture without antibiotics selection, SNL DG5 feeder cells were treated with G418 and blasticidin separately and then cell viability was calculated using MTT experiment. Compared with original G418 resistance gene expression, the result shows our insulated transgene expression has higher expression stability (**Fig. 3B**) over long time period implying HS4 insulator could protect from transgene silencing.

### Stem cell marker expression on established feeder cell line

**Figure 4:**
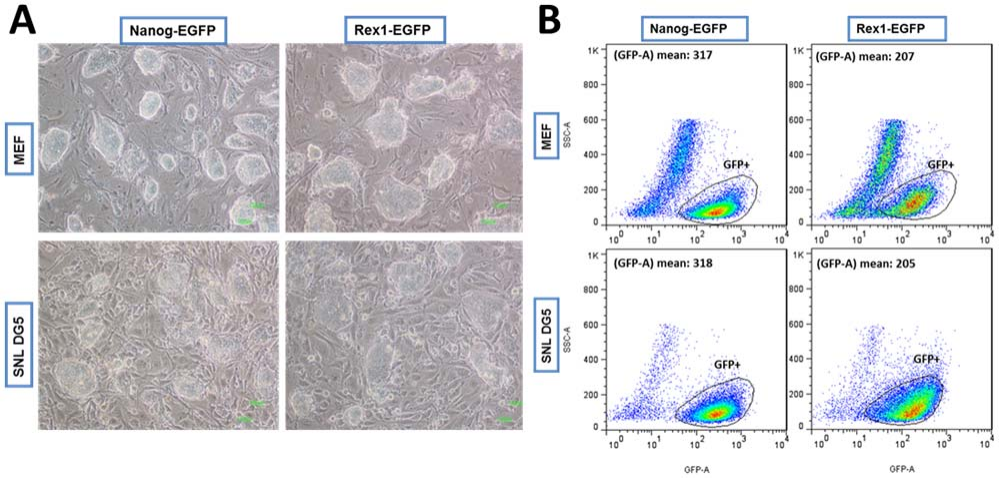
SNL DG5 feeder could maintain ES cell pluripotency. (A) Cell morphology of NG2S and RGOC ES cell line grown on MEF and SNL DG5 feeder layers. The colonies are shown by phase contrast microscopy with 10x objective, and the scale bar is 100 µm. (B) Flow cytometry analysis of two ES cell lines (Nanog-EGFP, Rex1-EGFP) after three passages cultured on MEF and SNL DG5 feeder layers.

To investigate whether the established SNL DG5 feeder cell line could maintain ES cell pluripotency, two pluripotency reporter ES cell lines, Nanog-EGFP and Rex1-EGFP were cultured on MEF and SNL DG5 feeder cells separately. After three continuous passaging, ES cell on both feeder cells still exhibited a typical ES cell morphology with tightly packed colonies and smooth boundary (**Fig. 4A**). Rex1 and Nanog driven EGFP expressions on both feeders were also measured with flow cytometry analysis. The data shows that the mean stem cell marker expression level of the MSCs cultured on SNL DG5 were indistinguishable from those with commonly used MEF feeder layer (**Fig. 4B**). These results indicate that our established SNL DG5 feeder layer could maintain the pluripotency of ESCs.

### Generation of stable ES cell clones with co-expressions of five fluorescence proteins

**Figure 5:**
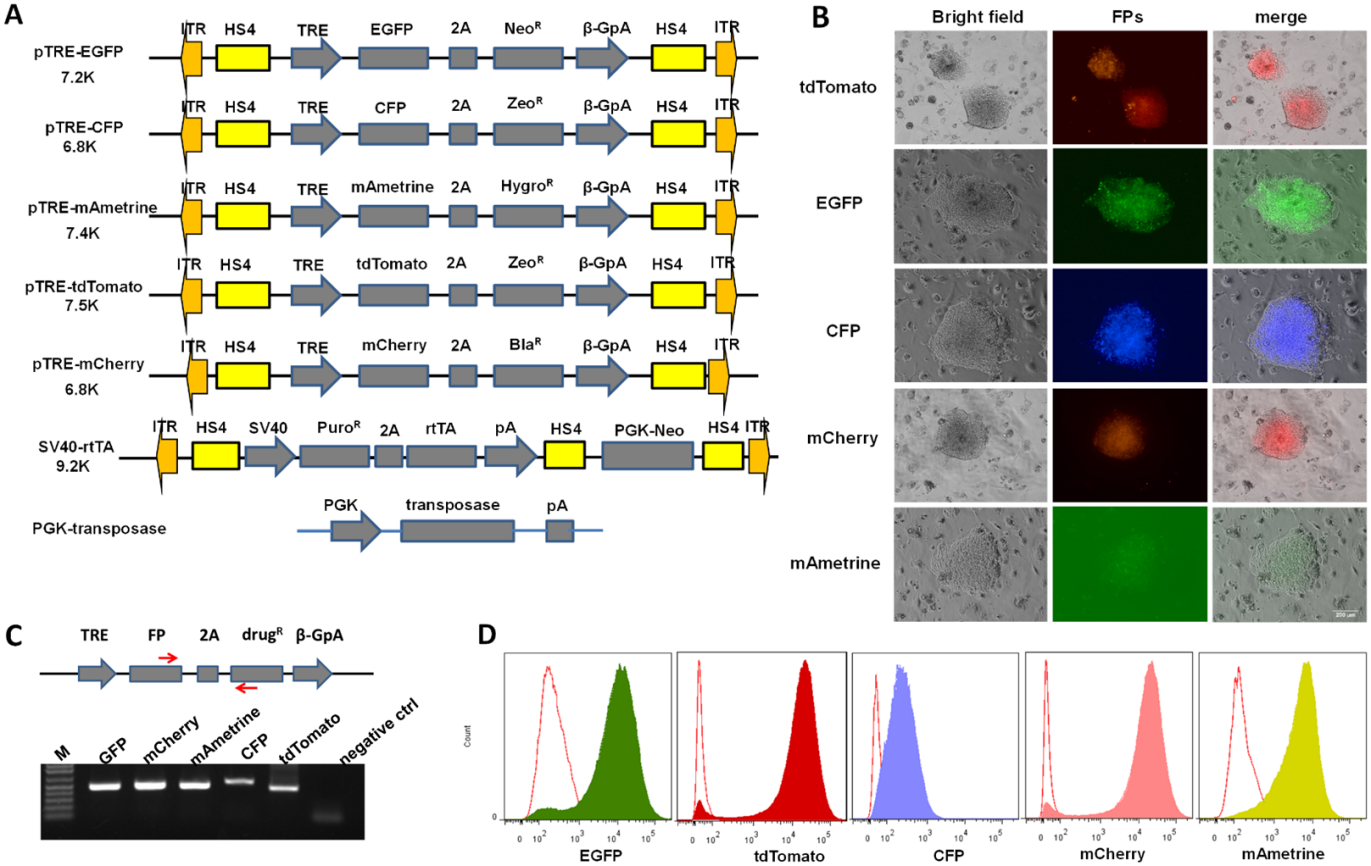
Generation of ES cell reporter cell line with multiple fluorescence proteins co-expression. (A) Schematic representation of inducible fluorescence protein expressing cassettes in the context of *PiggyBac* backbone. (B) Imaging of generated individual fluorescence reporter under different excitation light. Note that mAmetrine fluorescence was photographed under blue light thus has weak signal. The scale bar is 200 µm. (C) Genotyping analysis of generated ES cell reporter with five fluorescence proteins coexisted in single ES cell. (D) Flow analysis of generated No.12 multiple fluorescence protein stable ES cells. The thin red line on the left is the histogram without doxycline treatment as the negative control.

The establishment of SNL DG5 feeder cell indicates that *PiggyBac* system has very high transposition efficiency. To quantitatively measure *PiggyBac* mediated multiplex gene transfer in mouse ESCs, we generated five transgenes with inducible gene expression (TRE-FPs-T2A-drug^R^) on the context of *PiggyBac* backbone. We used flow cytometry analysis of single cells to identify simultaneous expressions of all five FPs. The TRE promoter was chosen for its tight regulation. We also utilized T2A peptide (*Thoseaasigna* virus 2A) to mediate the bicistronic translation of FPs and antibiotic resistance genes. Compared with the widely used internal ribosomal entry site (IRES), 2A peptide could mediate compatible expression levels of two or more proteins simultaneously [21], [28]. The detailed descriptions of the constructs were shown in **Fig. 5A**.

Although multiple fluorescent proteins have been used before to track cell fates [29], simultaneous expression of five different FPs have not been reported before to the best of our knowledge. Due to the various degrees of spectral overlapping among them, decomposition of real fluorescent protein intensities from instrumentation output must be carefully performed. Although it can be performed with software from microscope (e.g. spectral unmixing) or flow cytometry (compensation), the process is usually empirical and hidden. With our transposition systems, we would be able to establish a spectral unmixing process that provides quantitative assessments of the intensities of multiple FPs in single cell. We first used Ainv15 ES cell line [30] to generate individual ES cell lines to express the commonly used FPs (EGFP, CFP, mAmetrine, mCherry and tdTomato) individually. Since Ainv15 already contains rtTA transactivator, what we need to do is to transfect different TRE-driven FPs into this ES cell line. After antibiotics selection for about 1 week, stable clones were picked and propagated for future calibration experiments. In the absence of inducer doxycline, FPs will not be expressed. Once doxycline was added, due to the heterogeneity nature of tetON expression system, fluorescent intensities varied dramatically from one cell to another, which will help facilitate the computational analyses (**Fig. 5B**).

To generate multiple FPs expression in a single ES cell, we transfected the aforementioned five FPs carrying cassettes together with transactivator expressing plasmid (**Fig. 5A**) as well as helper PGK-transposase plasmid. Totally seven plasmids (six plasmids were to integrate into the chromosome) were mixed and transfected into CCE ES cell line. After antibiotics selection, we successfully isolated stable ES cell clones harboring six plasmids integration simultaneously. Genotyping indicated all six plasmids have been integrated into the chromosome (**Fig. 5C**). After doxycline induction, the FP expression was first observed under fluorescence microscopy using different channel. Flow cytometry analysis further confirmed that after doxycline treatment, the histograms of five FPs showed significant shift compared with the control indicating high transgene expression (**Fig. 5D**).

### Spectral unmixing of multiple fluorescence proteins ES cell reporter

**Figure 6:**
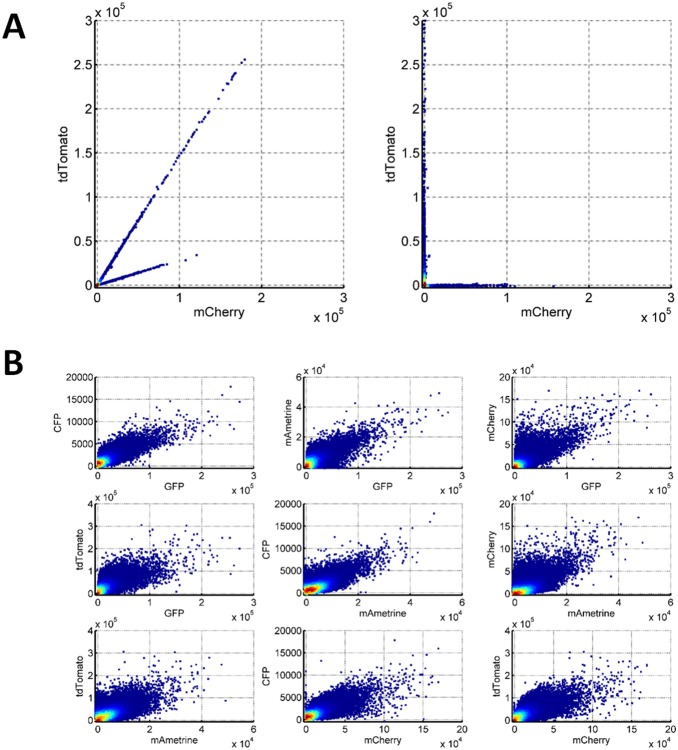
Spectral unmixing and verification of multiple transgenes in single ES cell clone. (A) Example of linear unmixing for cell lines with single mCherry and tdTomato fluorescence protein. (B) Linear unmixing of multiple fluorescence proteins based on ES cell lines with different individual fluorescence protein.

As mentioned earlier, although we could observe co-expression of all five fluorescent proteins in single ES cell based on fluorescence microscopy and flow cytometry, partial spectral overlap among FPs could mask the actual transgene FP expressions. Spectral unmixing is necessary for validation. To this end, we established a systematic method including: 1) using cells with single FPs to determine the spectral overlap; 2) using fluorescent-labeled rainbow beads to calibrate the amplifications of each channel for every experiments; 3) using simple and robust linear algebra equation to compute. The details of the computation are the following. For each fluorescent detection channel *i* with different excitation laser and emission filter, the intensities for the *i*th detection channel, *F_i_* follows this rule,
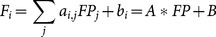
(1)where *FP_j_* represents the intensities of the jth FP, *a_i,j_* represents the fraction contribution coefficients of *FP_j_* to the ith detection channel, and *b_i_* represents the signal offset for the *i*th channel. Since we have established ES cells expressing individual FPs, the *a_i,j_* can be determined experimentally by flow cytometry analysis of all these lines. By using a matrix representation to simplify the equations, we could simply derive the equation to determine the intensities of each FPs in a cell,



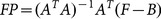
(2)Where *FP*, *F* and *B* are vector representations of *FP_j_*, *F_i_* and *b_i_*, and *A* is the matrix representation of the experimentally determined *a_i,j_*. To illustrate the effect of this method, we analyzed the two cell lines expressing mCherry or tdTomato, respectively, as shown in the left panel of **Fig.** **6A**, where there are significant signal interferences due to their emission wavelength overlapping. From the slopes of the two lines indicated in the figure, we could get the fraction contribution coefficient of the two FPs to the two fluorescent channels. By using all the relevant fluorescence detection channels and all five FPs, we determined the entire A matrix and B vector shown in equation 2. Based on equation 2 and the experimental determined parameters, we can easily compute the real FP intensities from signal intensities from each fluorescent detection channels. The results for mCherry and tdTomato cell line are shown in the right panel of Fig. 6A. The calculated mCherry and tdTomato signals are orthogonal to each other, meaning no interference.

With the methodology confirmed, we moved on to analyze one of ES cell clones (No. 12) carries all five FPs. All the FP expressions were induced in this clone by adding 1 µg/mL doxycyclin for two days. Then we analyzed their expression with flow cytometry with all the relevant fluorescence detection channels, and collected 20000 cells. Fluorescent data was extracted and analyzed with customized MATLAB (Mathworks, Natick, MA) code. Spectral unmixing process indicates the coexpression of all the FPs in single cells in this clone (**Fig. 6B**), which strongly confirmed the simultaneous integration of six plasmids (5 for FPs and 1 for rtTA) into ES cells with the *PiggyBac* transposition systems. In addition, this method also enable us to analyze the “intrinsic” *vs* “extrinsic” noises defined by *Elowitz* and coworkers that could help unveil the source of heterogeneity in gene regulatory systems [31].

### Transposition efficiency of *PiggyBac* mediated multiplex gene transfer in mouse ESCs

**Figure 7:**
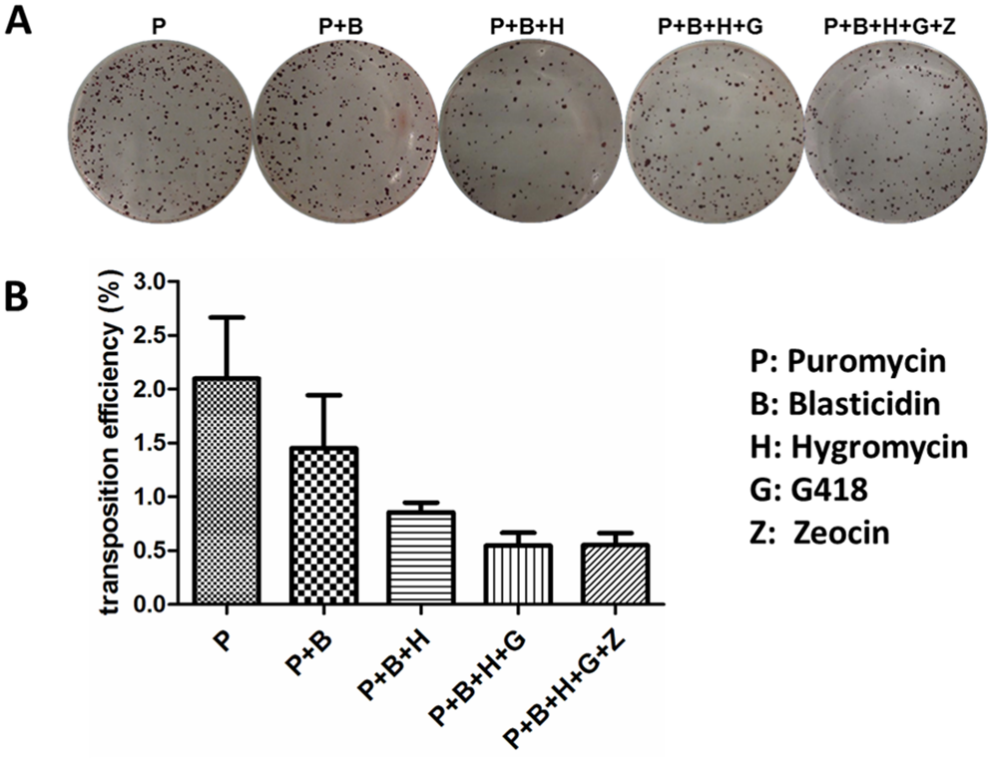
Transposition efficiency of multiplex gene transfer based on *PiggyBac* system in mouse ESCs. (A) AP staining for transfected stable ES cell clones using different combinations of antibiotics selections. Each dot represents a stable ES cell clone. (B) Transposition efficiency of *PiggyBac* mediated multiplex gene transfer. Data was based on two independent experiments.

In order to quantitatively measure the transposition efficiency with multiple transposons, we transfected seven plasmids into ES cells simultaneously, of which six plasmids were used for integration. One day after transfection, identical numbers of cells were re-plated in freshly prepared SNL DG5 feeders, and different types of antibiotics were added into culture medium for selection. Seven days later, the selected stable ES cell clones were fixed and stained with AP staining kit (**Fig. 7A**). AP-positive ESC clone numbers were calculated by the ImageJ software. Our AP staining experiment indicates the transposition efficiency was about 2% for a single transposon integration. However, transposition of different transposons is not independent since the frequency of transpositions of multiplex transposons was not significantly lower than that of a single transposon. The frequency of transposition of five transposons simultaneously only dropped slightly to about 0.5% when six transposons were cotransfected (**Fig. 7B**). These results demonstrate that the *PiggyBac* system is very efficient for multiplex gene transfer in mouse ESCs. To our knowledge, only one previous study reported *PiggyBac* mediated multiplex gene transfer (four transposons) in human cells but not mouse ESCs [32]. Our results indicate that *PiggyBac* system is a valuable tool for studying several genes at the same time in stem cell research. In addition, the transposon size in our study is between 7 kb–10 kb indicating even with relatively high cargo size, this *PiggyBac* still has high transposition efficiency.

## Discussion

Here, we have established an immortalized SNL feeder cell line which is resistant to multiple antibiotics simultaneously. Antibiotics resistance test indicates that it can display much higher resistance to concentrations of antibiotics commonly used for ES cell selection. ES cell culture showed that it can be used as a very good feeder layer for maintaining ES cell pluripotency and multiple antibiotics selection. This is the first feeder cell line with up to five antibiotics resistance genes coexist up to now. This will provide great flexibility when choosing appropriate antibiotics resistance feeder layers.

In mouse ES cell culture, the feeder layer can be replaced by adding LIF and other chemicals into growth medium but they are expensive. However, for human ES cell culture feeder layer still play critical roles due to the unknown factors secreted by MEFs. Therefore MEF is still widely used for ES cell culture in most laboratories. The commonly used MEFs are derived from 13.5 day embryo and can only be used within about four passages. New MEFs have to be isolated repeatedly which is labor and time intensive. Currently, there have been some substitutes for MEFs such as STO cell line (derived from a continuous line of SIM mouse embryonic fibroblasts) [33], 3T3 (evolved from 17–19 day mouse embryos) [34] and JK1 (an immortalized murine SMA^+^ CD34^+^ testicular stromal cell line) [35]. The immortalized STO cell line and its derivatives could be passaged indefinitely and has shown to be used as feeder layers in mouse or human iPS culture [5], [36]. SNL 76/7, first established by Allan Bradley and coworkers [3], is derived from the STO cell line transformed with neomycin resistance and murine LIF genes. However, in some ES cell experiments which multiple antibiotics need to be added for selection, the single antibiotic resistance SNL will not be suitable. Our established SNL DG5 feeder layer are therefore has great applications for future studies in which multiple antibiotics selection are needed.

Transposons are genetic elements which can move from one DNA locus to another by a “cut and paste” mechanism. Currently, many different types of transposon systems have been discovered and successfully applied into mammalian gene transfer experiments. The most widely used are Tcl-like transposon *Sleeping Beauty* (SB) [37], hAT-like *Tol2* [38] and *PiggyBac* (PB) [14]. Among the transposon mediated gene delivery methods, *PiggyBac* has been shown to have much higher efficiency in vertebrates and mammalian cells [25], [39]–[41]. Recently, Tucker *et al.* have successfully used *PiggyBac* to transfect four transposons into HEK-293 cell simultaneously [32]. However, the exact multiple gene transfer efficiency has not been conducted, especially in mouse embryonic stem cell. We first used four transposons to generate a pool of stable feeder cells which could support our ES cell selection experiment. Subsequently we increased the number of transposons to six in mouse ES cells. The results showed that even with up to six transposons. *PiggyBac* system still has about 0.5% transposition efficiency compared with 2% for single transposon transposition. On this regard, it also indicates that transposition mediated by *PiggyBac* system is not an independent event. It could be anticipated even with increasing number of transposons, *PiggyBac* system could still provide satisfactory transposition efficiency in most studies.

In summary, we have successfully established an immortalized SNL feeder cell layer for multiple antibiotics resistance. Furthermore, we characterized the transposition efficiency for multiplex gene transfer in mouse embryonic stem cell for the first time. The established feeder cell line will provide a great valuable tool for stem cell selection when two or more recombinant genes are introduced for biomedical research and drug development. And the high transposition efficiency using *PiggyBac* system would also enable us to study more genes simultaneously.

## Supporting Information

S1 Figure **Genotyping analysis of generated immortalized multiplex antibiotic resistance feeder cell lines.** The expected PCR product size for blasticidin, zeocin, hygromycin and puromycin resistance genes are 458 bp, 434 bp, 1082 bp and 659 bp respectively.
(TIF)
