# Supplementary figures and images for: PiggyBac Mediated Multiplex Gene Transfer in Mouse Embryonic Stem Cell
Source: PLoS One. 2014 Dec 17;9(12):e115072. doi: 10.1371/journal.pone.0115072 (PMC4269400; doi:10.1371/journal.pone.0115072)

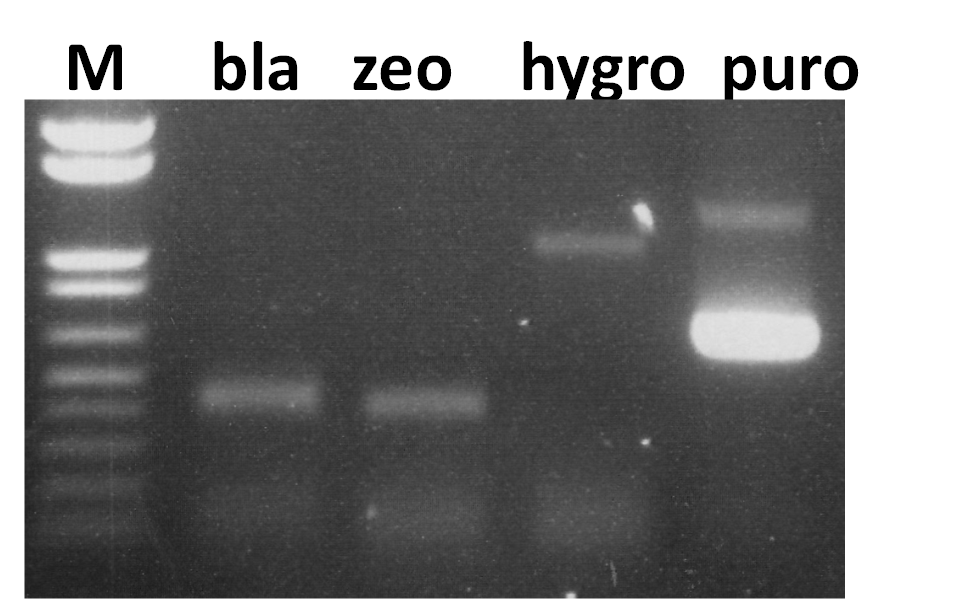

Supplement: S1 Figure — Genotyping analysis of generated immortalized multiplex antibiotic resistance feeder cell lines. The expected PCR product size for blasticidin, zeocin, hygromycin and puromycin resistance genes are 458 bp, 434 bp, 1082 bp and 659 bp respectively. (TIF) [file pone.0115072.s001.tif]
